# Association of Tramadol Versus Codeine Prescriptions with all-cause mortality and cardiovascular diseases among patients with osteoarthritis: a systematic review and meta-analysis of propensity score-matched population-based cohort studies

**DOI:** 10.1186/s42358-024-00417-4

**Published:** 2024-10-17

**Authors:** Mansour Bahardoust, Sepideh Mousavi, Maryam Zolfaghari Dehkharghani, Mahsa Arab, Heeva Rashidi, Habib Gorgani, Meisam Haghmoradi, Alireza Askari

**Affiliations:** 1 Department of Epidemiology, School of Public Health and Safety, Shahid Beheshti University of Medical Sciences, Tehran, Iran; 2 Pharmacy Faculty, Tabriz University of Medical Sciences, Tabriz, Iran; 3 Department of Health Care Administration and Policy, School of Public Health, University of Nevada Las Vegas (UNLV), Las Vegas, NV USA; 4 Department of Orthopedic Surgery, Faculty of Medicine, Golestan University of Medical Sciences, Golestan, Iran; 5 Bone and Joint Reconstruction Research Center, Department of Orthopedics, School of Medicine, Iran University of Medical Sciences, Tehran, Iran; 6 Department of Orthopedic Surgery, Urmia University of Medical Sciences, Urmia, Iran; 7 Present Address: Shafa Orthopedic Hospital, Baharestan Square, Tehran, 1157637131 Iran

**Keywords:** Osteoarthritis, All-cause mortality, Cardiovascular diseases, Tramadol, Codeine

## Abstract

**Background:**

Today, the prescription of tramadol in patients with osteoarthritis (OA) has increased significantly, which can be associated with serious consequences. Contradictory results have been reported regarding the association of tramadol versus codeine with the risk of all-cause mortality (ACM) and cardiovascular diseases (CVD).

**Methods:**

This systematic review and meta-analysis aimed to evaluate, for the first time, the association of tramadol versus codeine with the risk of ACM and CVD in OA patients for the first time. We searched PubMed, Scopus, Embase, Web of sciences, and Google Scholar with specific keywords and mesh terms to find relevant studies until January 2024. Two independent researchers did the process of searching and screening articles. Cochran’s Q and I2 tests evaluated the heterogeneity of the studies. Egger’s test was used to evaluate the existence of publication bias.

**Results:**

Seven population-based cohort studies, matched by the propensity score method, including 1,939,293 participants, were reviewed. The study pooled results did not show a significant association between the prescriptions of tramadol versus codeine with increasing the risk of ACM in OA patients. (Hazard ratio (HR): 1.084, 95% confidence interval (95%) CI: 0.883, 1.286, P: 0.56) In addition, the prescription of tramadol versus codeine was not associated with an increased risk of CVD in OA. (HR: 1.025, 95% CI: 0.89, 1.16, P: 0.68, I^2^ = 37.8%)

**Conclusion:**

Our systematic review showed that tramadol prescription compared to codeine in OA patients was not associated with an increased risk of ACM and CVD.

## Background

Osteoarthritis (OA) is one of the most important health problems worldwide, and its burden is expected to increase significantly with the increase in life expectancy and the aging population [1–3]. OA with cartilage destruction can lead to severe pain and impaired mobility [4]. Previous studies have reported that OA negatively affected 303 million people worldwide in 2017 [2, 5]. In addition to pain and negative effects on physical performance, osteoarthritis can be associated with several other outcomes, including mental health, sleep, work participation, and mortality [6–9].

Various treatments are recommended for OA, but only some of these drugs have adequate safety and efficacy [10–12]. Tramadol was recommended as one of the weak opioid agonists for the treatment of OA in 2013 by the guidelines of the American Academy of Orthopedic Surgeons, and in recent years it has been commonly prescribed for the treatment of OA [13]. According to the included studies, tramadol prescription has increased from 1 per 10,000 in 2013 to 11.4 per 100,000 (nearly 12 times) [14].

A number of studies have reported the association between tramadol use and serious complications, including mortality, venous thromboembolism (VTE), and CVD, in patients with OA [15–17]. However, conflicting results have been reported regarding the relationship of tramadol to codeine to increase the risk of mortality and CD in OA patients [17–19]. Xie et al. in a population-based cohort study, showed that using tramadol compared to codeine significantly increased OA patients’ risk of ACM, VTE, and CDV [15]. While Li et al. in 2022, did not report a significant association between the increased risk of ACM and CVD due to tramadol use versus codeine [16]. Therefore, the association of tramadol versus codeine with serious and life-threatening complications in OA patients is still unclear.

Therefore, in a systematic review and meta-analysis for the first time, we investigated the Association of tramadol vs. codeine as a milder and safer opioid prescription with the risk of ACM and CDV in OA patients. This regular review’s results can help doctors prescribe safer patient treatment.

## Methods

In this systematic review and meta-analysis, all population base cohort studies evaluated the Association between the use of tramadol Vs. Codeine was included with the risk of all-cause mortality and cardiovascular diseases in patients with osteoarthritis. This systematic review has been registered in PROSPERO (registration number CRD42024512781) and conducted based on the criteria of the PRISMA checklist [20].

The case group included patients with OA who had taken tramadol for at least 1 month. The control group included patients with OA who had taken codeine for at least one month with different doses.

### Methods for literature search

We first specified the keywords related to the research question based on the PICO strategy. The related mesh terms were specified for each keyword. Then, the Overall search strategy of the study was determined. To find relevant studies, we searched four databases: PubMed, Scopus, Embase, Web of sciences, and Google Scholar until January 2024. Each database was searched based on a special search strategy. Two independent researchers carried out the search process. The last search update was on January 12, 2024. The mesh terms were determined by the PICO (population, intervention, comparison, and outcome) format. The search was conducted using the following mesh terms.

((‘’ Mortality’’ OR ‘’Death Rate’’ OR ‘’ Mortality Rate’’ OR ‘’Death’’ OR ‘’ all-cause mortality’’ OR ‘’ all-cause”)) AND ((‘’ Cardiovascular Disease’’ OR ‘’ Major Adverse Cardiac Events’’ OR ‘’ Cardiac Events’’ OR ‘’ Cardiac Event‘’ OR ‘Adverse Cardiac Events’’ OR ‘’ CVD’’)) AND ((‘’Tramundin’’ OR ‘’Biodalgic’’ OR ‘’Jutadol’’ OR ‘’Nobligan’’ OR ‘’Tramadol”) OR (‘’ Morphinan-6-ol, 7,8-didehydro-4,5-epoxy-3-methoxy-17-methyl-, (5alpha,6alpha)-‘’ OR ‘’ N-Methylmorphine’’OR ‘’N Methylmorphine’’OR ‘’ Isocodeine’’ OR ‘’ Codeine Phosphate’’ OR ‘’ Ardinex’’)) AND (‘’ Osteoarthritides’’OR ‘’ Osteoarthrosis ‘’ OR “Degenerative Arthritides’’OR ‘’ Degenerative Arthritis’’ OR ‘’ Arthrosis’’ OR’’ Arthroses’’ OR ‘’ Osteoarthrosis Deformans’’).

### Eligibility criteria and data extraction

Population-based cohort studies by examining the association of tramadol Vs. Codeine use with the risk of all-cause mortality and cardiovascular diseases in osteoarthritis patients, studies with a follow-up of at least 12 months, and the estimation and reporting of the study’s effect size as the criteria for entering this review. A systematic meta-analysis was defined. Exclusion criteria included studies examining the effect of tramadol versus other opioids or stronger compounds derived from codeine, such as Oxycodone or Hydrocodone, examining tramadol versus no-use tramadol, examining the effect of tramadol versus codeine in populations without osteoarthritis, non-English language studies, Letters to the editor articles, case report studies or case series reports, review articles and meta-analyses, laboratory or animal studies, genetic studies and lack of access to the full text of the article.

**Fig. 1:**
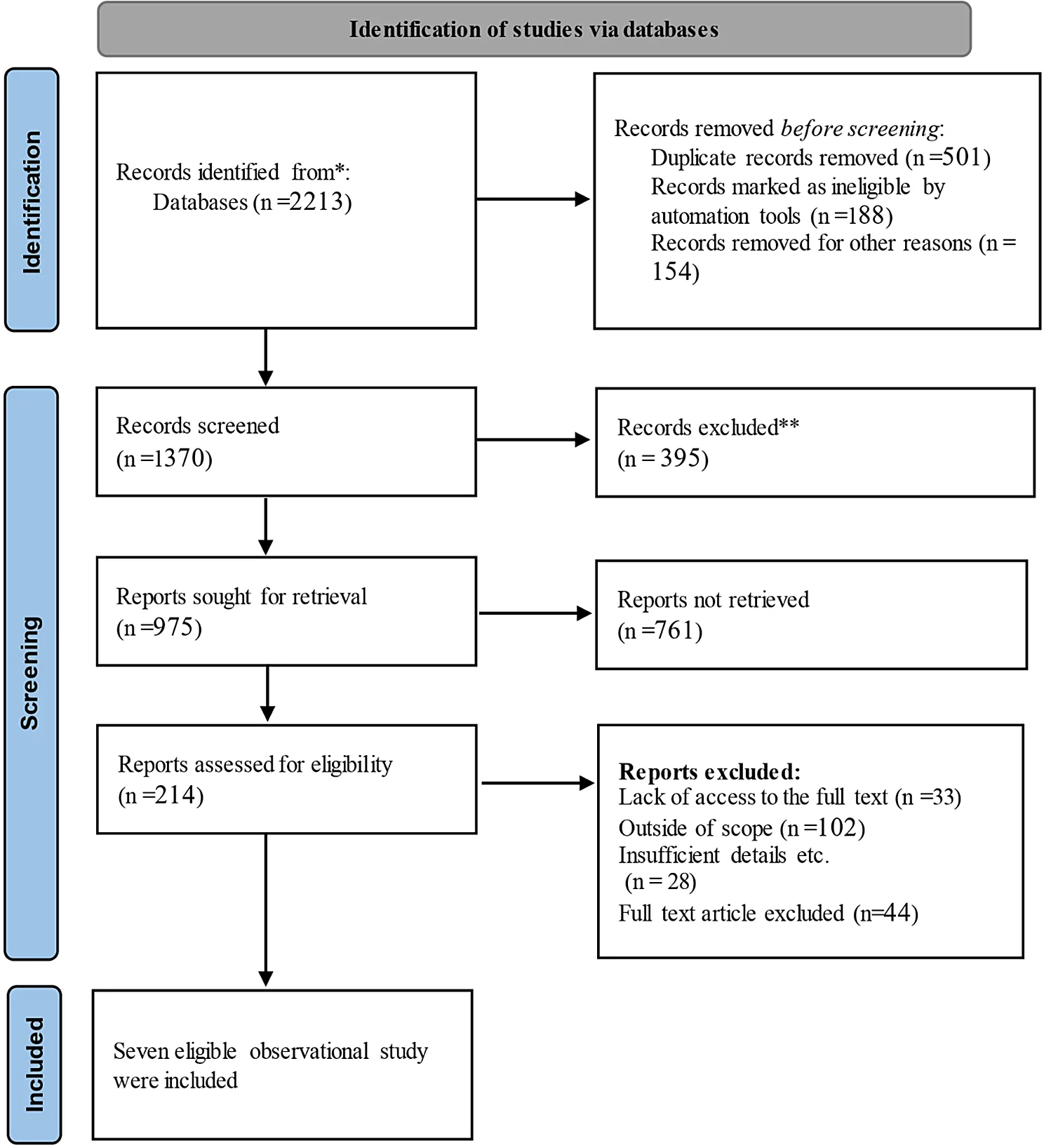
Flowchart of screening and inclusion of studies

Searching PubMed, Scopus, Web of Sciences, and Google Scholar databases, 2213 related articles were found. The articles’ titles, authors, and abstracts were extracted with Endnote version 20 software. Using EndNote software, 501 common and duplicate articles were detected and removed after combining the search results of four databases. In the initial screening of the search, 975 articles were selected to check their relationship with the research question. The screening used the title and abstract of studies to find studies related to the research question by two independent researchers. The process of cleaning articles was done to find relevant articles. The full text of 214 studies was read completely to extract the variables. Finally, seven population-based cohort studies were included in this systematic review (Fig. 1).

The required variables and data were extracted from the studies using a checklist based on the questions and objectives of this systematic review and entered into an Excel file. The variables examined and extracted in this systematic review include study authors, year of publication, population under study, type of study design, type of statistical analysis, methods for control confounding variables, mean age, gender distribution, setting of the study, the total number of people examined, the number of participants examined in the tramadol and codeine groups, overall mortality, the rate of all-cause mortality and cardiovascular diseases in each group per 1000 people, underlying diseases, history of diabetes, average body mass index, mean follow-up, the risk of all-cause mortality and cardiovascular diseases based on the Hazard ratio (HR) effect size in the 95% confidence Intervals (95% CIs) and the quality of studies in terms of risk of bias. In the data extraction process, if there is a difference between two researchers about a variable, a third researcher was used to resolve the difference.

### Quality assessment of included studies

In this systematic review, the Newcastle-Ottawa Quality Assessment Form for Cohort Studies checklist was used to assess the quality of studies regarding the risk of bias [21]. This checklist evaluates and scores the quality of studies included in regular review studies in the three dimensions: selection, comparability, and outcome/exposure. This checklist’s score range is between 0 and 9; a higher score means higher quality. The entered studies are based on quality scores in one of three categories: good, fair, and poor. They are classified as follows. Good (3 or 4 scores for the selected dimension and one or two stars for the comparability dimension and 2 or 3 stars for the outcome/exposure dimension), Fair (2 scores for the selected dimension and one or two stars for the comparability dimension and 2 or 3 stars for outcome/exposure dimension) and Poor (0 or 1 score for the selected dimension and 0 stars for the comparability dimension and 0 or 1 star for the outcome/exposure dimension).

### Statistical analysis

The extracted data were analyzed using Stata 17 software. The characteristics of the participants included in the study were reported using descriptive and mean statistics. The risk of mortality and cardiovascular diseases in all studies was extracted with the Hazard ratio (HR) in the 95% confidence interval (95% CI) to reduce heterogeneity. To control the effects of the study sample size, the pooled effect size was estimated with a random model. The Cochran Q and I^2^ tests were used to evaluate the heterogeneity of the studies. Egger’s test was used to evaluate the presence of publication bias. Due to the absence of publication bias for any of the outcomes in different studies, there was no need to use trim and fill analysis to solve publication bias. The risk of tramadol versus codeine use with ACM and CVD was reported using the pooled HR with a 95% CI.

## Results

This meta-analysis examined seven population-based cohort studies [15–19, 22, 23], which Matched with the propensity score method, including 3,649,890 participants, to investigate the risk of tramadol versus codeine with ACM and CVD. The mean age of tramadol and codeine users was 63.24 ± 9.88 and 64.11 ± 10.21 years, respectively. 57.2% of the participants were female. Based on the study evaluation checklist, all seven studies were good quality. Six studies investigated the prevalence of diabetes, which was 16.1%. Characteristics of participants, statistical analyzes, and quality of included studies are reported in Tables 1 and 2.

**Table 1:** Statistical analyzes and characteristics of the participants in the studies

Author	Year	Study design	Sample size	*N* of in tramadol group	Outcome	Mean Age (Year)	Sex (Female)	Statistically analysis
Zeng et al. [17]	2019	Prospective cohort	88,902	16,922	ACM&CVD	70.1	61.2%	Compared cause-specific mortality in the tramadol cohort with codeine, it matched the comparator cohort using a cause-specific Cox-proportional hazard model.
Wei et al. [19]	2020	Prospective cohort	66,048	42,722	CVD	68.3	61.8%	Comparison risk of CVD in the tramadol cohort with codeine matched comparator cohort using a cause-specific Cox-proportional hazard model adjusted for other variables.
Li et al. [18]	2020	Prospective cohort	56,325	7813	ACM&CVD	68	62.7%	Sequential propensity score-matched cohort study. Comparing the risk of ACM and CVD in the tramadol cohort with codeine, it matched the comparator cohort using a cause-specific Cox-proportional hazard model adjusted for other variables.
Xie et al. [15]	2021	Prospective cohort	3,330,572	28,206	ACM&CVD	53.1	51.7%	The possibility of confounding by indication was controlled by using propensity score matching. In addition, sensitivity analysis was performed to control for confounding variables, and the effect size was reported as HR in 95 CI.
Sørensen et al. [23]	2022	Prospective cohort	56,325	7813	ACM&CVD	68	62.7%	A pre-post design was used to evaluate changes in ACM and CVD after use of tramadol vs. Codeine.
Gau et al. [22]	2022	Prospective cohort	1320	880	ACM&CVD	NA	NA	Matching for age, gender, and year of diagnosis of rheumatoid arthritis was used for two groups. A Cox proportional regression model estimated future CVD development’s adjusted hazard ratio (aHR). Kaplan-Meier and log-rank tests were used to estimate the cumulative incidence of EC and CVD.
Li et al. [16]	2022	Prospective cohort	56,325	14,622	ACM&CVD	68	62.7%	Logistic regression was used to estimate propensity scores for the initial tramadol prescription. The basic characteristics of the tramadol group were compared with the pumpkin group before and after adjusting the propensity score. SAS software was used for statistical analyses, and hazard ratios (HRs) with 95% confidence intervals (95% CI) for the risk of tramadol versus codeine use with outcomes were calculated and reported.

**Table 2:** Distribution of underlying diseases, place of study, and quality of studies

Author	Diabetes (%)	Mean follow-up (years)	Country	Quality of studies
Zeng et al. [17]	16.1%	6.7	USA	Good
Wei et al. [19]	14.5%	0.49	USA	Good
Li et al. [18]	NA	0.98	British/Columbia/Canada	Good
Xie et al. [15]	9.7%	0.96	Spain	Good
Sørensen et al. [23]	13.1%	NA	Denmark	Good
Gau et al. [22]	11.5%	0.38	Taiwan	Good
Li et al. [16]	30.1%	0.99	British Columbia, Canada	Good

### The association of tramadol vs. codeine use with ACM

**Fig. 2:**
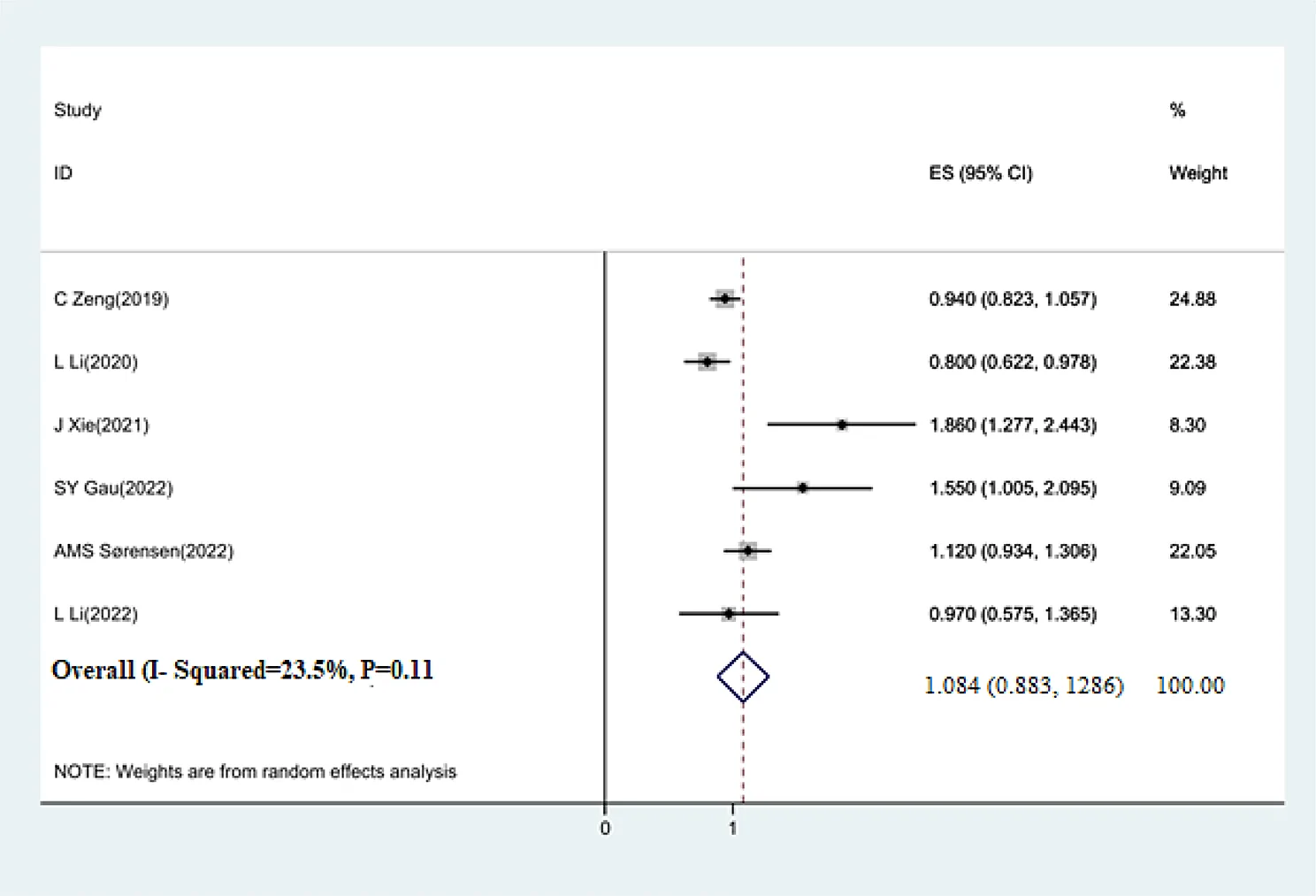
Pooled estimation of the effect of tramadol Vs. codeine on ACM

**Fig. 4:**
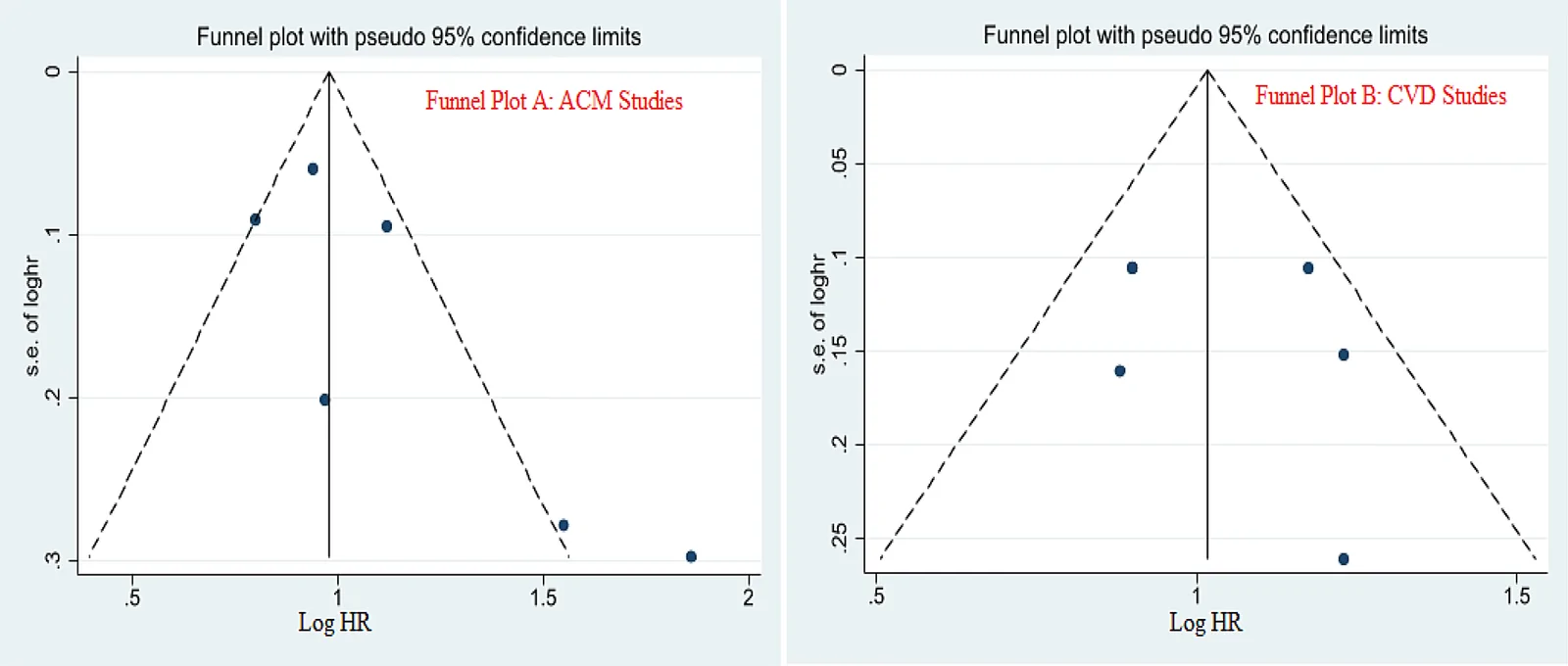
Funnel plots for evaluation of publication bias of studies based on the outcome

Six studies investigated the association between tramadol use ns codeine and ACM risk in patients with OA. The pooled result showed that although tramadol versus codeine was associated with an increased risk of ACM in osteoarthritis patients, this relationship was not statistically significant. (HR: 1.084, 95% CI: 0.883, 1.286, P: 0.56) (Fig. 2) The results of pooled studies had acceptable heterogeneity. Egger’s test analysis did not show statistical significance for publication bias in studies investigating tramadol use’s association with ACM. (Egger test: 2.52, p: 12, 95% CI: −1.02,6.07) (Fig. 4- funnel A).

### The association of tramadol vs. codeine use with CVD

**Fig. 3:**
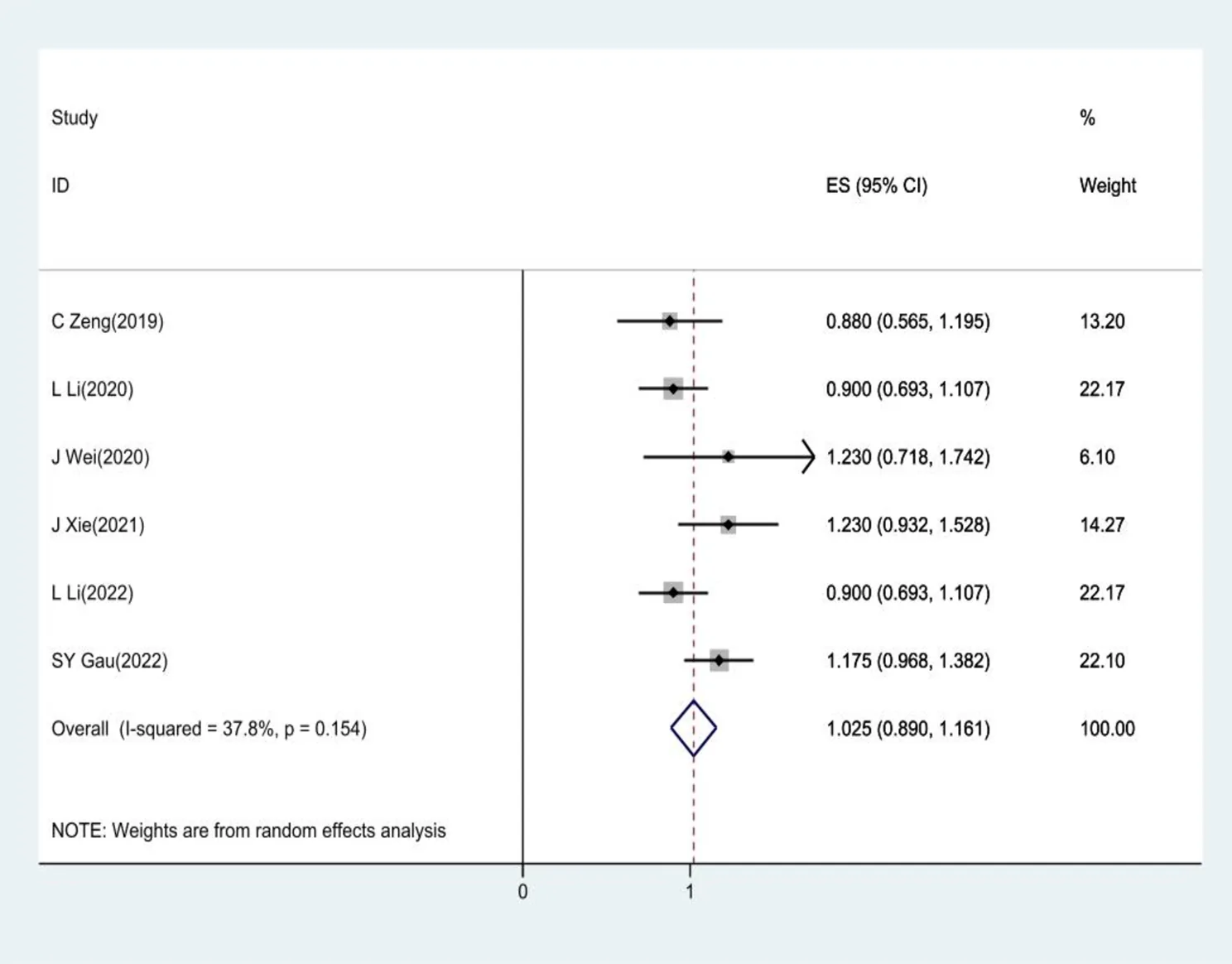
Pooled estimation of the effect of tramadol Vs. codeine on CVD

A number of six studies investigated the effect of tramadol versus codeine on the risk of CVD in patients with OA. The pooled estimate showed no statistically significant association between the use of tramadol and codeine with increased CVD risk. (HR: 1.025, 95% CI: 0.89, 1.16, P: 0.68, I^2^ = 37.8%) The included studies had lower heterogeneity. (Fig. 3) Egger’s test analysis did not show a significant estimate for the publication bias of the studies. The publication distribution of the studies is shown in Fig. 4, funnel plot B. (Egger test: 2.56, p: 0.53, 95% CI: −4.15, 6.91)

## Discussion

In the last decade, tramadol prescription for OA pain relief has increased significantly. A number of previous studies have shown an association between tramadol use and an increased risk of mortality, CVD, Venous thromboembolism (VTE), and fractures. Although most studies have been conducted in a cohort with a suitable sample size, conflicting results have been reported regarding tramadol versus codeine with the risk of mortality and CVD in OA patients. In this systematic review and meta-analysis study, for the first time, we evaluated the association of tramadol vs. codeine prescriptions in rheumatic patients with a risk of ACM and CVD. This meta-analysis did not show a significant association with the increased risk of ACM due to the use of tramadol compared to codeine in patients with OA. Also, this study did not show a significant relationship with the increased risk of CVD due to the use of tramadol compared to codeine in rheumatic patients. Based on the risk of bias assessment checklist, all included studies were of good quality. A number of the target population studies were complete patients with OA. In several studies, the relationship between the risk of ACM and CVD using tramadol versus codeine was measured in the subgroup of patients with OA. Xie et al. A population cohort study shown although the use of tramadol versus codeine was significantly associated with an increase in the risk of ACM and CVD in the sub-group of patients with OA, this increased risk was significantly lower in the subgroup of patients with OA compared to the total population examined in these cohorts. (HR: 1.86 Vs. HR: 2.31)

The pooled results of this meta-analysis, in which the included studies had adequate heterogeneity, did not show a significant relationship between increasing the risk of ACM and CVD with tramadol versus Codeine in patients with OA. Cepeda et al. [24] by examining the effect of oral tramadol prescription on physical performance, its duration of effect, and the safety of tramadol in people with OA in a systematic review, showed that the use of tramadol significantly reduced the symptoms, intensity of pain and improved the functional performance of these patients. In their study, they did not report any serious complications from tramadol use. In 2019, April et al. [25] examined the benefits and side effects of oral tramadol or tramadol with acetaminophen or NSAIDs in people with OA in a systematic review by examining 22 Randomized controlled trial (RCT) studies, most of which were of moderate quality, showed that the number of severe and serious side effects caused by tramadol use was very low and acceptable. The limitation of their study was the review of RCT studies with small sample sizes, short-time follow-up, and special conditions. In contrast, only population-based cohorts with high sample sizes and long-term follow-up periods were examined in our study. In another review study, Mansour et al. [26], by examining the safety of opioids, including tramadol in the management of OA on 17 RCT studies, confirmed the significant safety and tolerability of opioids to reduce pain in OA patients. In their study, the follow-up periods were short, and the studies were of moderate quality. Still, they did not report any serious side effects from using opiates, such as tramadol, to control pain in their patients. Based on our knowledge, the relationship between the use of tramadol versus codeine or other NSAIDs with the risk of ACM and CVD has not been investigated in any review study, so we could not discuss and compare the results of our study with other studies.

Our study had strengths and weaknesses that should be noted.

Weaknesses: In this systematic review, we examined population-based cohort studies in which the use or non-use of tramadol or other opioids, the dose, and the duration of use were recorded only based on patient self-reports. In addition, the cohort studies included in this meta-analysis selected different populations with different characteristics, which may affect the overall results. In addition, the use of tramadol in the studies included in this meta-analysis was self-reported, which can affect the final effect. In different studies, Codeine and tramadol were used in different doses, and also the follow-up length of the studies was different, which can have a negative effect on the results.

Strengths: Assessing the association of tramadol versus codeine with the risk of ACM and CVD in propensity-matched population-based cohort studies, in a systematic review and meta-analysis with good quality studies, for the first time were the most important strengths of this study.

## Conclusion

Our systematic review showed that tramadol prescription compared to codeine in OA patients, was not associated with an increased risk of ACM and CDV. Maybe this is due to comparing tramadol with an opioid (codeine). Limited studies have examined the association of tramadol use and non-use with the risk of ACM and CVD, and due to the limited number of studies, we were unable to examine the risk of ACM and CVD with non-tramadol users.

## Data Availability

The datasets used and/or analysed during the current study are available from the corresponding author on reasonable request.

## Abbreviations

OA: Osteoarthritis
ACM: All-Cause Mortality
CVD: Cardiovascular Diseases
VTE: Venous Thromboembolism
NSAIDs: Non-steroidal anti-inflammatory drugs
HR: Hazard ratio
95% CI: Confidence Interval
RCT: Randomized controlled trial
